# Application of Single-Cell Assay for Transposase-Accessible Chromatin with High Throughput Sequencing in Plant Science: Advances, Technical Challenges, and Prospects

**DOI:** 10.3390/ijms25031479

**Published:** 2024-01-25

**Authors:** Chao Lu, Yunxiao Wei, Mubashir Abbas, Hasi Agula, Edwin Wang, Zhigang Meng, Rui Zhang

**Affiliations:** 1 Biotechnology Research Institute, Chinese Academy of Agricultural Sciences, Beijing 100081, China; bararayung123@163.com (C.L.); weiyunxiao@caas.cn (Y.W.);; 2 Key Laboratory of Herbage & Endemic Crop Biology, Ministry of Education, School of Life Sciences, Inner Mongolia University, Hohhot 010070, China; 3 Cumming School of Medicine, University of Calgary, Calgary, AB T2N 4N1, Canada

**Keywords:** scATAC-seq, plant science, epigenomics, chromatin accessibility, cis-regulatory elements

## Abstract

The Single-cell Assay for Transposase-Accessible Chromatin with high throughput sequencing (scATAC-seq) has gained increasing popularity in recent years, allowing for chromatin accessibility to be deciphered and gene regulatory networks (GRNs) to be inferred at single-cell resolution. This cutting-edge technology now enables the genome-wide profiling of chromatin accessibility at the cellular level and the capturing of cell-type-specific cis-regulatory elements (CREs) that are masked by cellular heterogeneity in bulk assays. Additionally, it can also facilitate the identification of rare and new cell types based on differences in chromatin accessibility and the charting of cellular developmental trajectories within lineage-related cell clusters. Due to technical challenges and limitations, the data generated from scATAC-seq exhibit unique features, often characterized by high sparsity and noise, even within the same cell type. To address these challenges, various bioinformatic tools have been developed. Furthermore, the application of scATAC-seq in plant science is still in its infancy, with most research focusing on root tissues and model plant species. In this review, we provide an overview of recent progress in scATAC-seq and its application across various fields. We first conduct scATAC-seq in plant science. Next, we highlight the current challenges of scATAC-seq in plant science and major strategies for cell type annotation. Finally, we outline several future directions to exploit scATAC-seq technologies to address critical challenges in plant science, ranging from plant ENCODE(The Encyclopedia of DNA Elements) project construction to GRN inference, to deepen our understanding of the roles of CREs in plant biology.

## 1. Introduction

Plant tissues and organs comprise different kinds of cell types with highly specialized functions, making them more adaptive and resilient to various abiotic/biotic stress. The functional specialization of each cell type is in part attributed to cell-type-specific gene expression patterns governed by CREs(cis-regulatory elements) [1]. Notably, natural mutations associated with plant domestication and improvement further highlight the critical roles played by CREs in regulating agronomically important trait variation [2,3,4]. In maize, the intergenic region has been found to harbor agronomic quantitative trait loci (QTL), while distal regions have been identified as the likely location of a few domestication loci that are believed to contain cis-regulatory elements (CREs) [5,6]. Interestingly, further genetic experiments have demonstrated that these genomic loci modulate the expression of their target genes in a cis-acting regulatory manner. Therefore, the identification and characterization of cis-regulatory elements and how they deal with various input signals are imperative to answer key biological questions.

Open chromatin is typically depleted of nucleosomes, providing the possibility of physical interaction between transcription factors (TFs) and regulatory sequences to initiate transcriptional machinery to medicate cell fate commitment and differentiation [7,8]. It is well known that active CREs are normally located in accessible chromatin regions(ACRs) [8]. To characterize CREs, different methods have been developed to profile chromatin accessibility at the genome scale. The Assay for Transposase-Accessible Chromatin sequencing (ATAC-seq) identifies open chromatin by using a hyperactive Tn5 transposase that inserts sequencing adaptors into accessible regions harboring potential CREs [9]. Compared with other chromatin accessibility assays like DNase-seq (DNase I hypersensitive sites sequencing), ATAC-seq requires a smaller number of cells, making it especially suitable for rare samples with limited cells [10]. However, this approach generates an average number of signals across a group of cells, potentially obscuring the intricate cellular dynamics and regulatory programs specific to individual cells. The need to dissect the complexity and intercellular variability of chromatin accessibility has directed researchers toward single-cell techniques. The development and refinement of single-cell ATAC-seq (scATAC-seq) have the ability to address these limitations by profiling DNA accessibility at cellular resolution [11]. With the advancement of scATAC-seq, researchers can now dissect the heterogeneity of chromatin states among single cells within a population, gaining a more holistic insight into the gene regulatory networks (GRNs) and epigenetic mechanisms governing cellular identity. This technology has been widely applied in biomedical science, ranging from immunology [12,13] and stem cell developmental biology [14,15] to tumorigenesis [16,17], and it has been gradually expanded to the field of plant developmental biology [18,19,20].

In this review, we provide an overview of the recent progress in single-cell ATAC-seq and highlight its applications in plant biology, aiming to provide guidance for future studies conducted in the field of plant science.

## 2. scATAC-seq Unravels Epigenomic Regulatory Mechanisms Underlying Cellular Heterogeneity

To date, many single-cell transcriptome (scRNA-seq) studies have been performed in an attempt to answer some of the complex biological questions that exist in plants, providing cellular-level insights into the molecular mechanisms of plant development and response to environmental stresses. Multiple studies have demonstrated that various biological processes, including somatic embryogenesis and organogenesis, are tightly governed by multi-layer regulatory networks [21,22]. Accumulating evidence indicates that epigenetic mechanisms are an important layer in the regulation of gene expression through chemical modifications such as histone modification and DNA methylation, affecting the chromatin state at specific sites [21]. Moreover, several studies have documented that the transcriptional regulation of gene expression is not only governed by promoters but also by distal regulatory elements such as enhancers located at very long distances from the target genes, which features cell-type-specific functionality and dictates cell fate specification. Chromatin accessibility and higher-order conformational changes have an impact on the recruitment of specific transcription machinery and the relative spatial positions of functionally related CREs, which in turn affect the expression of adjacent genes [23]. Thus, the application of merely scRNA-seq alone cannot fully unravel the complexity of biological events; a combination of scRNA-seq and epigenomic characterization is required to bridge the gap.

### 2.1. Advances of Resolving Cellular Heterogeneity in Plants Using Traditional Bulk Assays

Differential accessibility in CREs, which underlie cell identity and functionality, contributes to cell-type variations through transcriptional programs [8]. CREs in chromatin-accessible regions are in a permissible state, allowing for physical access to TF binding sites to be gained to regulate gene expression spatiotemporally [8]. Therefore, uncovering the dynamics of the chromatin landscape will shed light on the complex gene regulatory mechanisms in plants.

Efforts have been made to isolate specific cell types from plant tissues in order to characterize the chromatin accessibility landscape either by manual dissection or fluorescence-activated cell sorting (FACS) or laser-capture microdissection (LCM). LCM is a technique where single cells or specific cell types can be isolated under the microscope from tissue sections for a variety of downstream applications, including RNA-seq, ATAC-seq, and other high throughput sequencing-based assays. LCM has been optimized for a wide range of plant species, allowing for the dissection of cells from paraffin sections that still retain histological details. In order to study the variance in photosynthetic efficiency between C4 and C3 leaves, LCM was employed to isolate various cell types at different developmental stages and domains in maize, with the goal of identifying regulators of early maize leaf development [24]. The application of LCM in woody plants is challenging due to the presence of high levels of suberin in cell walls. To address this problem, a streamlined protocol for LCM of specific cell types followed by RNA purification was developed to perform RNA-seq from the desired cell types [25].

A more refined method to profile chromatin accessibility for a specific cell type has recently been developed, termed INTACT-ATAC-seq. This protocol capitalizes on the isolation of nuclei tagged in specific cell types (INTACT), where nuclei in a given cell type are enriched by magnetic beads, followed by ATAC-seq library construction and sequencing, making it highly amenable to explore chromatin dynamics for specific cell types in plants. Several studies have applied this approach in plant science and confirmed its reliability to obtain the cell-type-specific chromatin landscape, as well as high-quality nuclei [26,27,28,29,30]. Another florescent sorting-based method named FACS-ATAC-seq is a technique where FACS is used to specifically purify fluorescence-tagged protoplasts from a mixed cell population, where a group of tagged protoplasts is lysed to release the nuclei for ATAC-seq. FACS-ATAC-seq has been successfully employed to characterize cell-type-specific chromatin accessibility in *Arabidopsis thaliana* inflorescence meristem, where high-quality results were achieved, as indicated by the quality control metrics in ATAC-seq [31].

However, these techniques still suffer from some problems in their application in plant biology, especially for non-model plant species. For LCM, specific cell types are dissected from tissues based on the microscopic inspection of cellular morphological characteristics. However, LCM requires stringent procedures to preprocess samples, as well as highly complex and sophisticated equipment. For the application of FACS in plants, samples need to be handled carefully as this involves enzyme digestions to isolate protoplasts, which can be time-consuming for some woody plants. In addition, both FACS and INTACT require prior knowledge of tissue-specific or cell-type-specific marker genes to mark specific cell types in a tissue, which are not applicable to most non-model plant species because marker gene information is rare and unavailable. In addition, both methods are dependent on the generation of stable transgenic lines, hindering their extension to those plant species recalcitrant to genetic transformation. Interestingly, recent bulk ATAC-seq assays in plants have shown that little variations in accessible regions have been observed across tissues, conditions, and even samples with different genetic backgrounds. One possible explanation for this phenomenon is that true cell-type-specific accessibility might be masked by cellular heterogeneity in bulk assays [32]. In conclusion, while these aforementioned techniques are able to profile chromatin accessibility at the cellular level by capturing specific cell types from tissues, they only partially mitigate the limitations which arise in bulk assays, meaning that they are still unable to resolve the cell-to-cell variations which occur within cell types due to the prevalence of sub cell types, rare cell types, and intermediate state cells.

### 2.2. scATAC-seq Profiles Revolutionize Our Understanding of Cellular Heterogeneity

**Figure 1:**
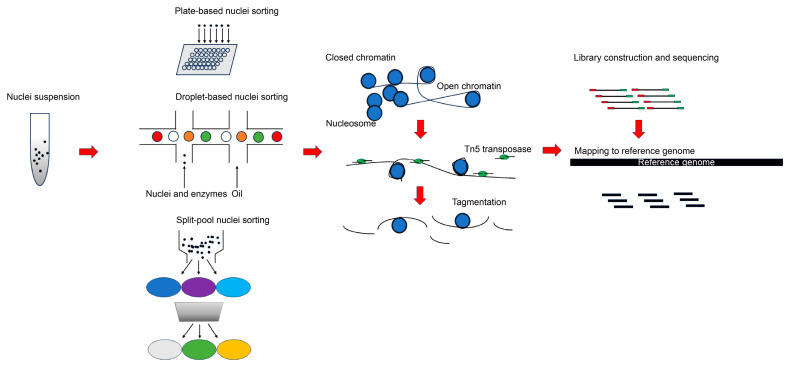
Major scATAC-seq experimental pipelines using different nuclei sorting protocols. This starts with obtaining high-quality nuclei suspension from tissues, nuclei sorting to ensure the encapsulation of each nuclei and reagents into separate droplets or wells for a subsequent transposition reaction, tagmentation of open chromatin with hyperactive Tn5 with sequencing adapters, the amplification of enriched accessible regions using PCR (Polymerase Chain Reaction) and sequencing, and mapping to the reference genome to capture genome-wide open chromatin regions.

Single-cell ATAC-seq is initially developed to capture the open chromatin of tissues at the cellular level using a prokaryote-derived Tn5-transposase, which features preferential insertion into accessible chromatin with short sequencing adaptors [33]. Currently, there are three types of experimental platforms for nuclei sorting and barcoding in scATAC-seq, namely plate- or array-based [11], droplet-based [34], and split-pool-based [35,36] solutions (Figure 1). Chromatin accessibility affects a wide range of developmental and physiological processes in plants, allowing for the recruitment of specific gene regulators to target binding sites. One study of plant cell totipotency has revealed that chromatin accessibility at totipotency-related transcription factors is significantly altered upon the perception of the auxin signal [21], highlighting its regulatory roles in plant development.

scATAC-seq is a powerful tool used to identify cell-type-specific CREs that may be associated with diseases or agriculturally important trait variation loci, enabling the reconstruction of cellular differentiation trajectories in highly heterogeneous systems [20,34]. ACRs captured by scATAC-seq are enriched in CREs typically bound by specific transcription factors [37]. Motif enrichment at differentially accessible regions enables the inference of potential upstream regulatory genes and the delineation of cell-type-specific gene target regulatory networks when coupled with paired expression data. Genetic variations in non-coding regions have an impact on a wide range of biological processes, such as transcription factor binding, histone modification, and mRNA translation. Similarly, in plants, sequence variations identified in several distal CREs are closely related to phenotypic variations attributed to the domestication of *Zea mays* [6,38,39,40]. Furthermore, changes in the flowering time of *Arabidopsis thaliana* suggest the existence of distal CREs and their regulatory roles in plant development [41,42,43,44]. Additionally, almost half of the heritable variance in quantitative traits in *Zea mays* is attributed to single-nucleotide polymorphisms (SNPs) in ACRs [45].

Cell-specific gene expression is the prerequisite for lineage specification, which is achieved by interactions between promoters, enhancers, and other kinds of CREs. Enhancers exhibit highly cell-type-specific histone modification patterns. These CREs are significantly associated with cell-type-specific gene expression patterns and are functionally accessible in a cell-type-specific fashion [46]. Repressive histone modifications, such as the H3K27me3 modification, are closely related to reduced chromatin accessibility. On the contrary, the deposition of H3K27ac at the promoters shows enhanced DNA accessibility [47]. Similarly, scATAC-seq analysis on the root tips of rice demonstrates that reduced histone methylation is observed in ACRs, and these modifications exhibit cell-type-specific patterns [18]. In plants, most CREs typically reside in unmethylated regions, which are enriched with accessible chromatin and histone acetylation, irrespective of tissue origin [48]. Several lines of evidence have demonstrated that the active removal of DNA methylation at CREs contributes to distinct developmental events, including the ripening of tomatoes and the perception of abiotic stimuli [49]. A study on *Arabidopsis thaliana* indicated that there is a rather complicated relationship between DNA methylation and chromatin accessibility. The loss of merely one or two types of CG, CHG, or CHH (where H indicates any base except G) DNA methylation fails to alter the inaccessibility context in chromatin regions [50]. A recent scATAC-seq analysis of maize has shown that cell-type-specific ACRs, especially those located distal to genes (>2 kb), mostly have the characteristics of enhancers, as evidenced by self-transcribing active regulatory region sequencing (STARR-seq) [51,52,53]. The adjacent regions flanking those ACRs with enhancer activity are usually marked with active chromatin histone modifications. Moreover, a large proportion of distal ACRs overlap with LTR (long terminal repeat) retrotransposons, including an agronomically important domestication locus, namely the TEOSINTE-BRANCHED 1-enhancer. These LTRs overlapping with ACRs have much lower levels of DNA methylation compared to those inaccessible LTRs. Interestingly, greater cell-type specificity is observed for ACRs co-localized with LTRs, implying potential functions in cell type determination [20]. A recent functional genomics study on tomatoes employed the CRISPR-Cas9 technique to target a CRE, obtaining various allelic variations surrounding a SNP associated with fruit weight. Notably, a significant increase in fruit weight was observed among various allelic variations [54]. Therefore, based on single-cell ATAC-seq technology, an in-depth mining of the spatiotemporal map of chromatin accessibility at the cellular resolution helps to reveal the core regulatory elements for plant growth and development, which can be further utilized to genetically engineer specific regulatory elements and boost agronomic productivity.

## 3. Challenges for Application of scATAC-seq in Plants

scATAC-seq is a powerful tool used to map the epigenomic landscape of complex organs at single-cell resolution and has been widely applied in animal science. However, the application of scATAC-seq in plants faces several challenges. Nuclei isolation is key to constructing high-quality libraries, and a few reports have been made on nucleus isolation methods in plant scATAC-seq. Most of these methods have been developed for immature or young organs in model plants, such as the root [19,55,56], inflorescences [57], and shoot apex [58]. A recent report on maize prepared the scATAC-seq library for six organs by nuclei isolation from fresh or flash-frozen tissue [20].

The lack of analytical tools is another obstacle which hinders the application of scATAC-seq in plants. It is well known that most bioinformatic pipelines for scATAC-seq are tailor-made for humans and mice, with parameters specifically optimized based on empirical and computational estimations from animal genomic features [59]. Moreover, many tools only include built-in databases for mammals, making the creation of custom databases for plants a time-consuming and technically challenging task [60]. Additionally, cell type annotation in scATAC-seq is more challenging compared with scRNA-seq, primarily due to the fact that the relationship between noncoding genomic regions and cell identity has not been fully elucidated in plants.

Lastly, the captured accessible regions need to be accurately mapped to a reference genome for the discovery of open or closed regions in a given cell [61]. Therefore, a well-annotated genome is essential for downstream analyses. High-quality genome assemblies can more accurately determine the locations of ACRs. Poor genome assembly can lead to incorrect mappings, which may compromise peak calling and annotation. In this section, the challenges associated with applying scATAC-seq are discussed, and recent advancements in addressing these challenges are summarized.

### 3.1. Preparation of Nuclei Suspensions Compatible with scATAC-seq

An aspect critical to the success of scATAC-Seq assays is the isolation of high-quality nuclei in sufficient amounts while maintaining the integrity of their overall structure throughout the experiment to prevent DNA from degradation. High-quality nuclei suspension is typically characterized as a lower clump rate and intact nuclear membrane. The presence of a nuclei clump is likely to interfere with cell/nuclei counting statistics, clog the chromium chip, and result in the failure of the GEM (Gel Bead in Emulsion) in a 10x Genomics microfluid system. The native architecture of chromosomes may also be disrupted by improper mechanical handling or endonucleases cleavage in broken nuclei, leading to the inaccurate enrichment of open chromatin. Therefore, the establishment of reliable nuclei isolation protocols is important for scATAC-seq.

Currently, several well-established protocols have been proposed, tailor-made for nuclei isolation from frozen or fresh samples in mammals for scATAC-seq [62,63,64,65,66]. However, only a few of the documented methods have been specifically designed for nuclei isolation in plants, most of which are used for bulk assays and have not been evaluated for their applicability in single-cell omics analysis [67]. Unlike animal cells, plant cells are coated with rigid cell walls and extremely abundant in various secondary metabolites, especially in some woody plant species like poplar. These characteristics can strongly interfere with nuclei isolation and possibly inhibit follow-up Tn5 transposition [68]. Hence, how to effectively remove these impurities remains problematic for scATAC-seq in plants.

**Figure 2:**
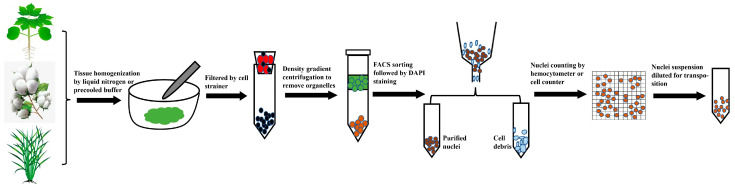
General procedure for nuclei isolation for scATAC-seq in plants. Fresh or frozen tissues are homogenized in pre-cooled nuclei isolation buffer or liquid nitrogen; the crude nuclei suspension is filtered using cell strainer; crude nuclei suspension is loaded on upper layer of density gradient to remove cell debris and organelles; nuclei are stained by DAPI (4′,6-diamidino-2-phenylindole) and further purified by FACS-based sorting; purified nuclei are then counted and diluted to proper concentration for library construction.

Recently, there have been several reports applying scATAC-seq in plant science, where different nuclei isolation protocols compatible with scATAC-seq have been developed [19,20,55,57,58,69,70,71,72]. Briefly, isolation protocols often involve laborious tissue homogenization, filtering the slurry through a cell strainer, and nuclei sorting by flow cytometry, and these protocols often require optimized chemical usage and centrifugation conditions for specific organs or species (Figure 2). The initial step for crude nuclei isolation varies among protocols, where tissues are homogenized either by chopping with a sharp razor blade or grinding in liquid nitrogen. Crude nuclei can also be obtained by protoplast lysis, but this may not be applicable to some plant tissues recalcitrant to protoplasting. Notably, the application of FACS is a routine step in nuclei isolation procedures, where there is an intention to separate intact nuclei from impurities like cell debris [20,55,57,58,69,71]. Whether or not to exploit FACS in plant nuclei isolation remains an open question. Recently, a handful of researchers have developed FACS-free nuclei isolation methods suitable for snATAC-seq (Single-nucleus ATAC sequencing)/snRNA-seq (Single-nucleus RNA sequencing), which generate high-quality libraries, as indicated by major metrics [19,70,72]. Additionally, other studies have demonstrated that the use of FACS to sort nuclei depends on the diameters of the nuclei in a given tissue, where nuclei with a diameter < 30 μm can be purified without FACS, and several rounds of filtering using a cell strainer are adequate for nuclei isolation, with only nuclei with a diameter greater than 30 μm requiring FACS. In summary, to achieve optimal results in scATAC-seq, caution should be taken to select proper nuclei isolation methods and make adjustments where necessary for special plant species.

Maintaining nuclei integrity and reducing nuclei clump rates have always been key in nuclei isolation for scATAC-seq. However, the widespread use of detergents in nuclei isolation poses considerable challenges to nuclei quality as these chemicals can undermine the structure of the nuclei membrane. To tackle these challenges, formaldehyde fixation is introduced prior to organelle lysis with a detergent during nuclei isolation in plants, greatly decreasing the occurrence of nuclei clumps and mitigating nuclei membrane disruption caused by the detergent [72]. Formaldehyde fixation makes nuclei resilient to detergent washes; thus, it can efficiently remove contaminations from organelle DNA, leading to a reduced doublet rate and making FACS unnecessary.

### 3.2. Analytical Tools Compatible with Plants

Most analytical tools, like Cicero (version 1.20.0), snapATAC (version 2.0), Signac (version 1.12.0), and ArchR (version 1.0.1), have been developed for model species in mammals like humans and mice. These are not out-of-the-box tools; they require adaptation for plant species, which may not be user friendly for researchers with limited bioinformatics skills. Furthermore, most of these tools only include pre-defined databases for mammals and are not applicable to plant species. Building a custom database for plants is a time-consuming process that demands an extensive comprehension of related bioinformatic knowledge.

We have tested the application of Cicero (version 1.20.0), snapATAC2 (version 2.5.3), Signac (version 1.12.0), Seurat (version 4.4.0), and ArchR (version 1.0.1) on our cotton scATAC-seq data and complied a table to compare several analytical tools for scATAC-seq (Table 1). Briefly, these tools are all able to perform differential peak analysis, but they vary in terms of functions such as pseudotime analysis, motif enrichment analysis, and multi-omics integration, as specified in Table 1. However, some analytical tools may require additional databases and software packages to complete the relevant analysis. For instance, Signac (version 1.12.0) is employed to analyze scATAC-seq data for cotton, which is a non-model plant species. However, the Bsgenome package is required for Signac to perform motif analysis and transcription factor footprinting; however, for unknown reasons, it has not been successfully constructed. Additionally, the default parameters in these tools are optimized for mammals and require adjustments for plants. To date, only one toolkit, named Socrates (version 0.0.9), is available for scATAC-seq in plants, but only part of the analytical code is publicly available [20]. Therefore, there is an imperative need to create a tailored scATAC-seq bioinformatic framework for the plant science community.

Due to the diversity of the scATAC-seq platforms, be it droplet-based or split-pool-based ones, these tools cannot meet the requirements needed for all platforms to conduct data preprocessing analysis. For example, both cellranger and snapATAC2 (version 2.5.3) are only applicable to the 10x Genomics scATAC-seq platform, which lacks scalability. Furthermore, most analytical tools only address specific problems and do not provide end-to-end (from data comparison to downstream clustering and cell annotation) analysis. Finally, the latest single-cell sequencing technology can simultaneously perform multi-modality (multi-omics) characteristic analysis on the same set of cell samples. However, most current analytical tools can only analyze single-cell data of one modality and cannot analyze and integrate multi-omics data [77].

### 3.3. Challenges for Cell Type Annotation in scATAC-seq

The open chromatin regions identified by single-cell ATAC-seq mainly fall within non-coding regions. At present, there is a lack of databases related to cis-acting elements and cell type annotation in plants. It is challenging to directly annotate cell types based on differences in peak information. While the read count in promoter regions for known cell type marker genes can be calculated as the predicted value of associated adjacent gene expression, some studies have found that simple promoter accessibility is not an ideal predictor of gene expression. Additionally, for most non-model species, tissue and cell-type-specific marker gene databases are not available.

Determining which genes are highly specific to a given cell type is a commonly used strategy to mark unknown cell groups with a proper identity in single-cell omics analysis. The identification of marker genes with high cell-type specificity is therefore important for cell type annotation. The identification of market genes in scATAC-seq is relatively challenging as the features used in this assay are a set of genomic coordinates, which are highly dataset-dependent and make it hard to interpret and compare with different datasets. One solution to this problem is to calculate gene scores based on read counts from certain gene body and promoter regions, which results in a cell-by-gene matrix similar to scRNA-seq.

Presently, many analytical tools are available to identify marker genes for scATAC-seq, such as Signac (version 1.12.0), SnapATAC2 (version 2.5.3), ArchR (version 1.0.1), and SEMITONES (https://github.com/ohlerlab/SEMITONES, accessed on 22 January 2024). Most of these tools extract read counts from open chromatin regions with the gene body and promoter to calculate the gene activity score of accessible genes. Marker genes are then identified using a similar strategy in scRNA-seq, and cell type annotation is implemented by interrogating the chromatin accessibility at canonical marker genes. Unlike Signac (version 1.12.0), ArchR (version 1.0.1), and snapATAC2 (version 2.5.3) [60,78,79], which are specifically designed for scATAC-seq analysis, SEMITONES is more scalable as it can identify marker features from scRNA-seq, scATAC-seq, and even spatially resolved transcriptome in a cluster-independent manner [80]. SEMITONES implements the strategy of enrichment scores calculation to identify cell-type-specific peaks. As this is a well-known difficulty in cell type annotation when using marker peaks, the GREAT (Genomic Regions Enrichment of Annotations Tool) algorithm is introduced to assign significantly accessible peaks to nearby genes. Cell type annotation is then made possible by the assigned genes and their enriched GO terms [80]. SEMITONES was initially developed for human data and benchmarked with other tools to confirm its reliability and efficiency. Recently, this tool was applied to the scRNA-seq root atlas comparison between wild-type and cell type mutants in *Arabidopsis thaliana*, indicating its applicability to the plant science community [81].

The identification of marker features in most analytical tools is based on Euclidean distance determined by feature differences between the cell cluster of interest and other cell clusters. However, the marker features identified in these tools may not be reliable in some cases. Specifically, features highly enriched in a rather small portion of cells in a given cell group, e.g., less than 5% in a cell cluster, are still likely to be identified as marker features. To avoid this potential problem, a new method called COSine similarity-based marker gene identification (COSG) [82] was developed for the identification of marker genes based on a cosine value that is highly accurate and scalable for the identification of marker features. It has been proved to be applicable to scRNA-seq, scATAC-seq, and spatially resolved transcriptomes. COSG (version 1.0.0) excels in dealing with super large datasets and accomplishes marker gene or peak identification within a very short time frame [82]. In conclusion, the marker features identified by COSG (version 1.0.0) exhibit higher cell-type specificity compared with other existing approaches.

### 3.4. The Annotation Quality of Reference Genomes

The reference genome serves as a map against which the DNA fragments obtained from scATAC-seq are aligned, which helps to identify accessible regions in the genome. The quality of the reference genome will determine the accuracy of ACRs localization, which may impact downstream analysis and the interpretation of the results.

To evaluate the effect of reference quality on scATAC-seq analysis, we have compared different genome assemblies with varying levels of annotation quality in our dataset analysis. The results indicate that it has a strong impact on scATAC-seq in terms of QC(quality control) metrics, peak calling, and cell clustering. The 10x Genomics scATAC-seq tutorial also demonstrates that a high-quality genome reference is required to achieve desirable results and meet the QC metrics, which is also important for scRNA-seq. In a recent study applying scRNA-seq in non-model plants, high-quality reference genomes were first assembled to improve mapping rates, ensuring the accurate quantification of scRNA-seq [83]. Therefore, it is advisable to evaluate the quality of a genome assembly first to generate reliable results in scATAC-seq.

## 4. Current Strategies for Cell Type Annotation in scATAC-seq

**Figure 3:**
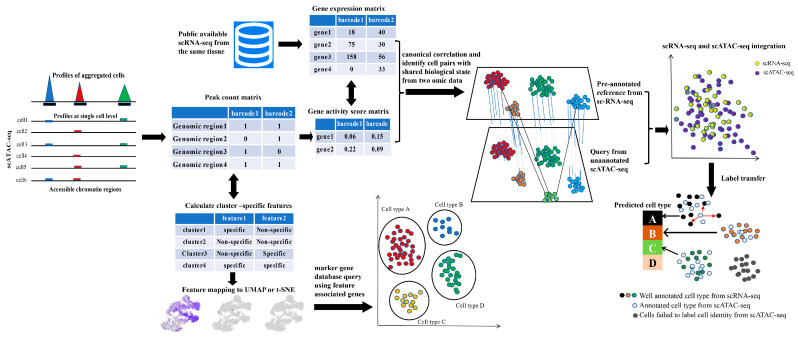
Major cell type annotation strategies in scATAC-seq. Basically, there are two ways to complete cell type annotation in scATAC-seq analysis. The first method is the conversion of peak count matrix to cell by gene matrix by calculating gene activity score. Marker genes for each scATAC-seq cluster are identified based on gene activity score, and the marker genes are subsequently used to query related marker gene database to assign proper cell identity to each cluster. Provided that scRNA-seq data for the corresponding tissues is available, co-embedding of both scATAC-seq and scRNA-seq in lower dimension map can assist in cell type annotation. Finally, based on differential peak analysis, other cluster-specific features are also helpful to cell type annotation in scATAC-seq, such as enrichment motif for each cluster or calculation of motif activity in each cluster.

The first step in interpreting single-cell omics data is to decipher cellular heterogeneity based on reliable cell type assignment. This enables us to gain a basic understanding of the types of cells that make up complex tissues and how the proportions of these cell types vary across different developmental stages or under various external stimuli. Identifying cell types inaccurately can lead to obtaining entirely false conclusions in subsequent scATAC-seq analysis. Therefore, cell type annotation plays a key role in scATAC-seq analysis. However, annotating cell types in single-cell ATAC-seq is especially challenging due to the limited availability of analytical tools and epigenomic databases for chromatin accessibility at the cellular level. Additionally, active ACRs captured by scATAC-seq are mostly located in non-coding regions, and the link between chromatin accessibility, cell functional specialization, and nearby gene expression remains elusive, making cell type annotation even more challenging. Generally, depending on the availability of scRNA-seq data from the same sample, cell type annotation can be performed using scATAC-seq alone or combined with scRNA-seq as a reference (Figure 3).

### 4.1. Cell Type Annotation Based on scATAC-seq Data

After cells pass quality control and aggregate into different clusters based on similar chromatin accessibility patterns, cell type annotation is performed based on differential peak analysis. In the 10x Genomic scATAC-seq cell type annotation tutorial, cells are simply assigned a specific cell type based on promoter accessibility at known marker genes, motif enrichment from cluster-specific peaks, or a combination of both using a series of logical expressions in a visualization software Loupe Cell Browser (version 6.0.0). However, recent studies have indicated that chromatin accessibility in the promoter region alone is not a perfect indicator of expression for associated genes. To solve this problem, the latest toolkits, like Cicero (version 1.20.0), Signac (version 1.12.0), and ArchR (version 1.0.1), employ a more advanced and refined algorithm to infer gene expression from scATAC-seq data and introduce a new feature termed gene activity score or gene score (GS) that show better consistency with gene expression, especially when integrated with scRNA-seq data.

The ArchR (version 1.0.1) toolkit benchmarked 50 GS models and selected the model that performed best under various test conditions [78]. Briefly, co-accessible sites to a gene’s promoter region are allocated a distinct weight in the calculation of GS so as to achieve better concordance with actual gene expression. In Cicero (version 1.20.0), it combines accessibility in a given gene’s promoter with linked distal sites’ accessibility and terms it as the gene activity score [59]. Considering that the expression values in RNA-seq data typically approximate log negative binomial distribution [84], the aggregated accessibility values are also exponentiated and scaled by the overall exponentiated gene accessibility values. Interestingly, in both cases, the GS was found to be positively correlated with corresponding gene expression and can be used as a proxy for gene expression in downstream cell type annotation. The calculation of GS for each cell generates a new feature matrix where each row represents a gene, and each column represents a cell barcode. All of these toolkits (Cicero version 1.20.0, Signac version 1.12.0, and ArchR version 1.0.1) require prior knowledge of cell-type-specific genomic regulatory elements to manually annotate cell clusters in a supervised manner. Genes with a GS that exhibit a cell-type-specific pattern are extracted as candidate marker genes, which are then searched against publicly available marker gene databases or references in the literature for cell identity assignment.

Given the exponential increase in cell numbers assayed in single-cell chromatin accessibility, the development of computational tools for automatic cell type annotation in scATAC-seq has garnered significant attention. Currently, there are several computational tools available for scATAC-seq cell type annotation, and benchmark analyses have been performed to evaluate these tools in terms of efficiency and robustness. These computational tools can be classified as supervised, semi-supervised, and unsupervised. The evaluation indicates that Bridge integration exhibits the best performance among these cell type annotation tools. EpiAnno (version 1.0.0), a cell type annotation framework, incorporates a Bayesian neural network to annotate scATAC-seq in a supervised manner. This analytical tool can identify cell-type-specific peaks and motifs, but it has some limitations, such as lower scalability for large datasets [85]. Cellcano (version 1.0.2) is another supervised cell type annotation tool, this tool is computationally more efficient and scalable for large datasets [86].

Apart from the above cell type annotation methods, other genomic features are also used for cell type annotations, such as the motif enrichment of transcription factors with known functions in a given cell type. Signac (version 1.12.0) employs two ways to perform motif analysis [60]. The hypergeometric test-based method is used to identify overrepresented motifs in differential accessible regions (DARs) from each cluster. An alternative way to perform motif enrichment analysis is to identify cluster-specific motif activity by computing a per-cell motif activity score using tools like chromVAR (version 1.1.1), which aims to capture motifs that are differentially accessible across different clusters [60].

### 4.2. Integration with scRNA-seq for Cell Type Annotation

Abundant scRNA-seq data with high-quality cell type annotations provide a valuable resource for cell identity assignment in scATAC-seq. Annotating scATAC-seq with scRNA-seq reference for cell type identification requires the data integration of these two modalities, which has been reported in several research works based on plants. Basically, the first step of data integration is to unify the features in each set of omics data in order to make them comparable with each other. For scATAC-seq, the peaks identified by the cell count matrix are converted to genes using the cell matrix through the calculation of GS. However, there are still many challenges faced when attempting to integrate single-cell multi-modal data, primarily due to data complexity, varied levels of sparsity, and the existence of a batch effect. To overcome these obstacles in data integration, several integrative analysis frameworks have been developed for single-cell multi-omics data integration. Seurat (version 4.4.0) implements a well-established algorithm, namely mutual nearest neighbor (MNN), to assign cell types from scRNA-seq to scATAC-seq using a label-transfer approach [87]. scJoint, a recently developed multi-omics integration tool, adopts a transfer learning method. It works in a semi-supervised manner and can efficiently integrate scRNA-seq and scATAC-seq data at an atlas scale by utilizing information from referenced scRNA-seq data [88]. The following label transfer and data visualization are carried out by implementing an internal neural network to train labeled and unlabeled data. Converting the scATAC-seq data into scRNA-seq data through GS calculation, however, is not free from problems, often resulting in a low-quality GS matrix and failure to accurately match the relation between two sets of omics data. To tackle this problem, another proposed integrative framework called scDART has recently been proposed, which enables simultaneous data integration for scRNA-seq and scATAC-seq and the learning of cross-modal relationships [89].

### 4.3. Cell Type Annotation in Non-Model Organisms

For non-model organisms, cell type annotation in scATAC-seq poses several challenges. Firstly, the lack of well-documented cell-type-specific marker genes presents a significant challenge. Secondly, scATAC-seq data, which measure chromatin accessibility, are highly sparse and noisy due to the low copy number of DNA fragments. The features in scATAC-seq are in the form of genomic coordinates, which cannot be directly translated to associate the gene expression for cell type annotations. Lastly, tissues with limited marker gene information often require the manual dissection of anatomical structures under a microscope and the isolation of specific cell types for de novo marker gene identification. These experiments demand researchers with a strong background in plant anatomy, making the process more challenging.

To date, several scRNA-seq studies have been reported in non-model plant species, including peanuts, poplars, tea trees, cotton, and *Brassica rapa* [57,58,59,60,61]. The first strategy employed for cell type annotation involved using gene annotations from the model plant *Arabidopsis thaliana*. Specifically, cluster-specific or differentially expressed genes were obtained using computational tools, and homologs of these genes in model organism were identified and queried against the plant marker gene database for cell type annotation. This method has proven to be effective for several non-model plant organisms, where most cell clusters were successfully assigned proper cell identities. The homolog gene annotation strategy also applies to scATAC-seq through the calculation of GS with a cell-type-specific pattern. However, some problems still exist concerning homolog genes in polyploid species, where gene functions may have undergone significant changes during long-term evolutionary history due to gene duplications. One potential solution is to filter out one-to-many homologous genes and consider only one-to-one genes as candidates for cell type identification [90,91,92].

Another possible solution to tackle cell type annotation in non-model organisms is cross-species comparison with well-annotated scATAC-seq datasets from the same tissue in model organisms. This strategy has the advantage of eliminating the need to find cell-type-specific marker genes. Seurat(version 4.4.0) has been widely used in scATAC-seq cell type annotations via label transfer from well-annotated scRNA-seq data in plants. Seurat (version 4.4.0) also supports cross-species data comparison from different modalities. From our perspective, it is very likely to integrate scATAC-seq datasets from non-model organisms with scRNA-seq data from model organisms to annotate cell clusters. Specifically, the peak by the cell matrix is converted to a gene by the cell matrix through GS calculation, and then, querying the homolog genes for the converted matrix unifies features in both model and non-model organisms, paving the way for data integration and cell type annotation. In mammals, cross-species comparison has been employed to annotate human lung cancer cells using scRNA-seq data from mouse lung cells. Interestingly, the results of this indicated that most cell groups were successfully assigned cell types with high confidence [93]. However, this strategy also faces some challenges due to the deep divergence across plant species. One of these problems is that not all tissues share cell-type-specific expression profiles. Fortunately, other layers of transcriptional regulation, such as TF binding motifs and their cognate TFs, tend to be evolutionarily conserved. scATAC-seq enables the identification of motif enrichment across different datasets, implying its great potential in this strategy.

## 5. Future Perspectives for Applying scATAC-seq in Plant Science

### 5.1. Refining Plant ENCODE Project at Single-Cell Level to Better Serve the Plant Science Community

Many plant species, especially model plants like *Arabidopsis thaliana* and *Oryza sativa*, have been completely sequenced and well annotated. The idea of the plant ENCODE (The Encyclopedia of DNA Elements) project was recently proposed, aiming to collect and standardize a plant epigenomic dataset for a wide range of plant species [94]. The project has been fueled by the great expansion of publicly available epigenomic data and related databases, like ChIP-Hub, which provides a comprehensive and ready-to-use web service for exploring plant gene regulatory networks [95,96]. Inspired by this philosophy, the RiceENCODE project has been initiated to build a comprehensive epigenomic database by collecting and reprocessing both publicly available epigenomic and transcriptomic data [97]. These projects have some limitations, as most of the data derive from bulk assays and largely overlook the impact of cellular heterogeneity on this information. Therefore, the emergence of scATAC-seq has provided the possibility of bringing the plant ENCODE project to a single-cell resolution.

ACRs characterized by scATAC-seq are the hallmark of CREs, some of which typically exhibit a cell-type-specific pattern and are putative enhancers. We anticipate that the updated plant ENCODE project will start with model plants as they have the most abundant annotation databases, bioinformatic tools, and analytical pipelines. The plant ENCODE project could include a cell-type-specific chromatin accessibility page where users can simply query either by cell type or gene ID to obtain basic information about ACRs in a given cell type or gene locus. Through integration with other types of bulk or single-cell-level epigenomic data, we can characterize how these ACRs covary with other epigenetic features like DNA methylation, histone modification, and spatial chromatin interactions, thereby providing a comprehensive epigenomic landscape at single-cell resolution.

### 5.2. Cross-Species Cell-Type Comparison Provides New Insights into Evolutionary Hierarchy

The exploration of cell development and molecular biology has been empowered by the wide application of state-of-the-art single-cell sequencing techniques, deepening our understanding of the heterogeneous nature of cell populations in a complex system and redefining the conception of cell identity. The wealth of single-cell sequencing data from multiple species enables the comparison of cell types across many species in terms of gene expression profiles and the epigenomic landscape, making it possible to unravel the cell lineage commitment and functional diversity from an evolutionary perspective [98]. The technology holds the potential to construct species phylogenic trees at cell type level, which can recapitulate the evolutionary trajectory between the same cell types from different species.

Cross-species comparison based on single-cell omics data is a powerful way to solve problems in biomedical science. Pigs are believed to be one of the best host species for human organ generation, which is fundamental to regenerative medicine. However, differences in organs between humans and pigs present a major challenge for breeding functional human tissues/organs in an animal host. scRNA-seq was employed to systematically compare epiblast development among pigs, humans, and monkeys. Cross-species comparison analysis at the single-cell level has revealed species-specific differences mainly derived from multiple factors, namely pluripotency progression, epigenetic and transcriptional regulations of pluripotency, and cell surface proteins. Therefore, cross-species analysis based on single-cell sequencing is a powerful way to delve into evolutionarily conserved and divergent pathways during mammalian development [99].

Inspired by research into mammals, several scRNA-seq studies in plants have also performed cross-species comparisons for specific cell types. These studies mostly selected the plant root as the input material for single-cell analysis, primarily due to its abundant marker genes and ease of protoplasting. By comparing gene expression patterns and development trajectories, functionally conserved and divergent cell types are identified, providing new insights into the evolutionary mechanisms contributing to cell type diversity in plants [20,83,100].

In addition, a recent study on the integration of pan-species cell type data reveals that there are molecular signatures across diverse growth conditions. Comparative translatome analyses of rice, tomato, and arabidopsis cell populations suggest that the expression conservation of root meristems is increased compared with other homologous populations. In addition, the functions of constitutively expressed genes are more conserved than those of cell type/tissue-enriched genes. These observations suggest that higher-order properties of cell type and pan-cell type regulation are evolutionarily conserved between plants and animals [101].

**Figure 4:**
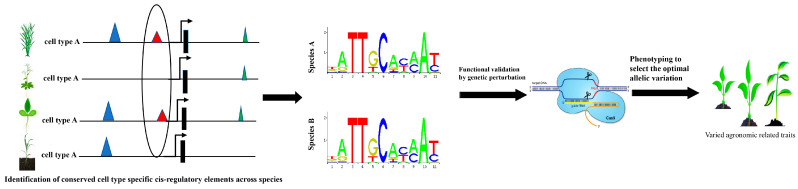
Identification of conserved cell-type-specific CREs and genetic variances beneficial to agronomic traits. Through integration of scATAC-seq from multiple species, peaks near the same genes are compared to identify conserved cell-type-specific CREs that may be essential for agronomic traits. Gene editing is then employed to target the regions of interest to validate its function in plant development and screen allelic variation beneficial to crop improvement.

In light of the success of cross-species analysis using scRNA-seq in plants, we believe that it is also possible to conduct analysis based on scATAC-seq; this shall be a more promising field to understand plant evolutionary history at the cellular level. The genomic distribution of ACRs identified by both scATAC-seq and bulk ATAC-seq reveals that most of them are in non-coding regions, which are important cis-regulatory sequences impacting target gene expression. Intriguingly, comparative genomics analysis has discovered conserved CREs proximal to genes in plant genomes, yet our knowledge of their functional relevance to cell identity and development remains poorly understood [102]. Several studies have indicated that these conserved non-coding sequences (CNSs) are mostly linked to genes with transcription and binding functions, as well as to syntenic genes and those originating from whole-genome replications, illuminating the critical role played by CNSs in driving genome evolution. We postulate that scATAC-seq might be a powerful tool to investigate the impact of CNSs on genome evolution through cross-species comparison. This cross-species analysis in scATAC-seq enables the identification of CNSs in ACRs between the same cell type across genetically distant organisms, as well as different cell types within the same organism, which may provide novel insights into cell biology and evolution (Figure 4). Through generating a repository of CNSs within the Solanaceae family, researchers have identified two highly conserved cis-regulatory sequences across different species and further validated their functional conservation by CRISPR-Cas9. Notably, CNSs have been experimentally confirmed to act as cis-regulatory regions. Moreover, genetic variation in long-range CREs contribute to gene expression divergence and ultimately affect agronomic traits. Therefore, these CNSs identified by scATAC-seq provide valuable resources for functional genomic studies. Though targeting to these CNSs by gene editing methods, a couple of allelic variation are obtained and mutations that are beneficial to crop improvement are selected.

In spite of the great advantages that they offer, cross-species comparisons have been challenged by complicated factors. The batch effects derived from technical variations also have a strong impact on both scATAC-seq and scRNA-seq integration for cross-species analysis [98]. Additionally, the evolutionary distance between ortholog and paralog genes should be well understood for proper cross-species data integration [98]. Lastly, poorly understood evolutionary forces, which arise from stochastic variations and selection, further complicate cross-species comparisons [98]. To circumvent these obstacles, various computational tools and sophisticated algorithms have been proposed for the cross-species integration and comparison of single-cell omics data. These analytical frameworks, like Seurat (version 4.4.0) and Signac (version 1.12.0), hold the promise of elucidating how evolutionary forces operate at single-cell resolution and trace the evolutionary origins underlying cell type diversity and developmental plasticity.

### 5.3. Toward Building a Cell Atlas through Multi-Omics Data Integration

The invention of microscopes and the advancement of state-of-the-art microscopic technology have substantially spurred the development of cell biology, allowing biologists to observe cellular structures in a finer resolution. Over the past centuries, scientists have endeavored to characterize and classify cell populations in heterogeneous tissues using both morphological and molecular signatures. Defining cell identity is relatively more reliable based on the expression profile of biological macromolecules such as RNA and protein, which provides uniform and unbiased results and does not require prior knowledge of cell morphology, which tends to be empirical.

Most plant species are multicellular organisms where cells are the basic units of tissues. A comprehensive catalog of cell types in plants and a deeper understanding of how plant cells work together to respond to external stimuli and mediate internal signaling are crucial to fundamental science in agriculture. Unraveling the heterogeneous nature of plant cells requires state-of-the-art technology to identify and map all the cell types in a given species and determine the position and organization of each biological molecule at the tissue or even single-cell level.

Considering the significance of identifying cell types and widely available single-cell sequencing data, the Plant Cell Atlas (PCA) project has recently been proposed, similar to the Human Cell Atlas (HCA) initiative [103]. The PCA project aims to use a combination of approaches to construct cellular reference maps for different plant species. These maps showcase detailed information defining the functionality of cell types in a given organ, including cell position, biological function, and major features (such as gene expression, DNA methylation, histone modification, and chromatin state) that distinguish them. It is anticipated that the establishment of PCA shall have an enormous impact on basic science in plants and provide novel insight into gene regulation in unprecedented detail and resolution.

### 5.4. scATAC-seq Intertwines with Lineage Tracing to Address Basic Questions in Plant Biology

Charting the developmental trajectory of terminally differentiated cell types is fundamental to stem cell biology. A detailed record of how cell states change over the course of organ development offers the opportunity to unravel the molecular mechanisms of cell fate determination during embryogenesis or other important biological processes. To study the developmental path of cell populations in complex tissues, various lineage-tracing methods have been developed to facilitate the mapping of these relationships among cell groups [104,105].

Several analytical tools can computationally infer cell developmental trajectories based on scATAC-seq datasets. Cicero (version 1.20.0) was initially developed to construct cis-regulatory networks and perform chromatin accessibility analysis [59]. After dimension reduction and cell clustering, cell trajectory analysis was performed for lineage-committed cell types. As Cicero (version 1.20.0) is built upon monocle, which has been widely used for constructing cell trajectory analyses and has proven its efficacy in plants, it is very likely to be adapted for scATAC-seq in plant studies. The CRE/LOX(causes recombination/locus of crossing (x) over, P1) lineage tracing technique, adapted from the mammalian system, has recently been applied to map stem cell differentiation history in plant science. For example, to determine the stem cell organizer in vascular cambium, a CRE/LOX-based lineage tracing system was established to understand how the procambium and pericycle in roots differentiate into vascular cambium and phellogen [106]. A recent report on pluripotency acquisition in callus employed the CRE/LOX system coupled with scRNA-seq to trace cell lineage in middle cell layers and confirmed its regeneration potential [107]. The middle cell groups were further defined as progenitor cells to infer the differentiation trajectory. The CRE/LOX system requires marker genes in a given cell type, which can also be obtained from scATA-seq by interrogating the chromatin accessibility at known marker genes. It is expected that the marker genes identified from scATAC-seq are readily available for the CRE/LOX system to trace cell development tracks and validate the computationally inferred cell differentiation trajectories. As this genetic cell tracing technology can position the progenitor in vivo, it is speculated that this system is well suited to answer questions about the origin of progenitors in explants during somatic embryogenesis in plants [21].

### 5.5. Gene Regulatory Network Analyses Using scATAC-seq Data

Gene regulatory networks (GRNs) are interpreted as graph models where transcription factors and their target genes interact with each other to form a topological structure [108]. The dissection of GRNs is of biological significance in the way that cellular identity specification is governed by these complex networks in a spatiotemporal manner.

Although several studies in plants have successfully used bulk omics data to infer GRNs [21,29], the results of bulk assays represent average measurements in a given sample and fail to resolve complex regulatory networks specific to cell types or cell states in a heterogeneous tissue. The rapid development of single-cell multi-omics technologies has made it possible to infer GRNs at the cellular level by the integration of expression and epigenomic information. A newly developed analytical framework named SCRIP can build single-cell-level GRNs based on a regulatory potential model [109]. It is implemented through the integration of scATAC-seq data with publicly available TF chromatin immunoprecipitation sequencing (ChIP-seq) data. This tool has its own advantage in that the combination of TF ChIP-seq data and motif references can overcome the inaccuracies of motif-based methods, resulting in enhanced precision in identifying cell-type-specific TF binding sites. Pairing scATAC-seq with scRNA-seq using computational approaches is another strategy to infer GRNs at the cellular level. Integration with scRNA-seq enables the association of distal cis-regulatory elements to genes, which forms the basis of TF-gene GRNs inference [110]. However, applying these tools to plant science is currently challenging, as most of them only support model mammalian species like humans and mice. Therefore, there is a pressing need to develop such tools for the plant community. We anticipate that the development of GRNs inference tools for scATAC-seq in plants will greatly enhance our insights into plant development.

## Figures and Tables

**Table 1:** Comparison of several scATAC-seq analytical tools.

Analytical Tools	Platform	Motif Enrichment	Doublet Removal	Pseudotime Analysis	Batch Correction	Upstream Analysis	Applicable to Other Omics Data	Multi-Omics Integration	Reported in Plants
Cicero	R	No	No	Yes	Yes	No	No	No	No [59]
snapATAC2	Python/R	Yes	No	No	Yes	Yes	Yes	Yes	No [73]
Signac	R	Yes	No	Yes	No	No	No	Yes	Yes [19]
ArchR	R	Yes	Yes	Yes	Yes	No	No	Yes	Yes [18]
SCALE	Python	No	No	No	Yes	No	No	No	Yes [74]
cisTopic	R	Yes	No	No	Yes	No	Yes	No	No [75]
epiScanpy	Python	No	No	Yes	Yes	No	Yes	No	No [76]

Note: Yes indicates that this tool is able to perform this analysis, while No means that it cannot be used for this function.
